# Meeting Aerobic Physical Activity Guidelines and Associations With Physical Fitness in Men With Metastatic Prostate Cancer: Baseline Results of the Multicentre INTERVAL‐GAP4 Trial

**DOI:** 10.1002/cam4.70261

**Published:** 2024-12-04

**Authors:** Lisa Umlauff, Stacey A. Kenfield, Robert U. Newton, Nicolas H. Hart, Fred Saad, Kerry S. Courneya, Rosemary Greenwood, Wilhelm Bloch, INTERVAL‐GAP4 Steering Committee/Coordinating Centres Members/Protocol Development Working Group Members, Moritz Schumann

**Affiliations:** ^1^ Department for Molecular and Cellular Sports Medicine, Institute of Cardiovascular Research and Sports Medicine German Sport University Cologne Cologne Germany; ^2^ Department of Urology and Epidemiology & Biostatistics University of California San Francisco California USA; ^3^ Exercise Medicine Research Institute, School of Medical and Health Sciences Edith Cowan University Joondalup Western Australia Australia; ^4^ Human Performance Research Centre, INSIGHT Research Institute, Faculty of Health University of Technology Sydney Sydney New South Wales Australia; ^5^ Cancers Génito‐Urinaires Centre Hospitalier de l'Université de Montréal Montreal Quebec Canada; ^6^ Faculty of Kinesiology, Sport, and Recreation, College of Health Sciences University of Alberta Edmonton Alberta Canada; ^7^ York Trials Unit, Health Sciences University of York York UK; ^8^ Department of Sports Medicine and Exercise Therapy, Institute of Human Movement Science and Health Chemnitz University of Technology Chemnitz Germany

**Keywords:** androgen deprivation therapy, androgen receptor inhibitor, endurance performance, maximal strength

## Abstract

**Background:**

This study compared the physical activity level of men with metastatic prostate cancer at baseline of the multicentre INTERVAL‐GAP4 trial to the American Cancer Society guidelines and examined associations with physical fitness.

**Methods:**

A total of 140 men on androgen deprivation therapy (ADT) were included in this cross‐sectional analysis of baseline data from the INTERVAL‐GAP4 trial. Exclusion criteria included a maximum of 1 h of vigorous aerobic exercise or one structured resistance exercise session per week but no restrictions on habitual physical activity. Moderate‐to‐vigorous physical activity (MVPA) was assessed using a modified Godin–Shephard Leisure‐Time Physical Activity Questionnaire. Physical fitness measurements included peak oxygen consumption (VO_2_peak), maximal power output (*W*_max_), 400 m walk time, one‐repetition maximum (1RM) of leg extension, leg press, chest press and seated row, and handgrip strength. Quantile regression was used to analyse associations of MVPA with physical fitness outcomes at the 25^th^, 50^th^ and 75^th^ percentiles of the physical fitness distributions.

**Results:**

Total self‐reported MVPA was 60 (IQR: 0, 180) min per week, with 29% meeting the aerobic physical activity guidelines. There was a statistically significant association of higher MVPA with higher relative VO_2_peak at the 25^th^ (*β* = 0.53, *p* = 0.020) and 75^th^ percentiles (*β* = 0.66, *p* = 0.001), relative *W*_max_ at the 25^th^ (*β* = 0.05, *p* = 0.003), 50^th^ (*β* = 0.05, *p* = 0.009) and 75^th^ percentiles (*β* = 0.07, *p* = 0.004) and reduced 400 m walk time at the 75^th^ percentile (*β* = −4.26, *p* = 0.023), with *β* corresponding to the change in the dependent variable for each one‐hour increase in weekly MVPA.

**Conclusion:**

Few men recruited to the INTERVAL‐GAP4 trial were meeting aerobic physical activity guidelines at baseline. Higher MVPA was associated with better aerobic capacity and walking performance but not maximal strength in men with metastatic prostate cancer on ADT.

**Trial Registration:**

ClinicalTrials.gov: NCT02730338; German Clinical Trials Register: DRKS00010310

## 1 Background

Physical fitness comprises a set of attributes related to the individual's ability to perform activities of daily living and engage in physical activity (PA) [1]. There is strong evidence that physical fitness can serve as a marker of health and predict morbidity and mortality in both healthy and diseased populations [2, 3, 4, 5, 6]. Two core components of physical fitness are neuromuscular and cardiorespiratory fitness. Improved neuromuscular fitness is associated with enhanced physical function, a lower incidence of metabolic syndrome and reduced mortality [7, 8, 9, 10, 11]. Furthermore, maintaining or even developing muscle strength at any age benefits the ability to perform basic activities of daily living, thus promoting independence [7]. Similarly, higher levels of cardiorespiratory fitness are linked to a lower risk of cancer, stroke and overall mortality [12, 13]. Importantly, the health benefits of higher neuromuscular and cardiorespiratory fitness are also evident in populations with pre‐existing disease [14], including cancer [3, 15, 16].

Regular PA can promote physical fitness by increasing muscle mass, cardiovascular health and overall physical function in all healthy adults, including those of older age [17, 18, 19, 20]. The term PA refers to any movements produced by the muscles that result in energy expenditure, whereas exercise is a subset of PA that describes planned, structured and purposeful activities with the aim to maintain or enhance physical fitness [21]. In individuals with cancer, both PA and exercise have been associated with improvements in physical function, bone health, cancer‐related fatigue and pain [22, 23, 24]. Based on these findings, specific recommendations regarding PA for cancer survivors have been issued. The American Cancer Society recommends that cancer survivors engage in regular PA, with the aim to achieve at least 150 min of moderate intensity PA per week [25, 26]. Despite this, several studies of individuals with lymphoma, head and neck, prostate, breast, colorectal and bladder cancer report that most cancer survivors fall well below this recommended level of PA [27, 28, 29, 30, 31, 32]. A potential reason for this is that individuals with advanced cancer experience a progressive disease burden that is often accompanied by physical symptoms such as pain, fatigue and a general decline of physical function, which limit their ability to be physically active [33]. In addition, side effects of cancer treatments may further increase morbidity and contribute to inactivity among individuals with advanced cancer. The American Cancer Society therefore acknowledges that the needs and benefits of PA may differ depending on the cancer stage [25].

Prostate cancer is the second most common malignancy in men worldwide [34]. While early stage prostate cancer is curable, many men with prostate cancer eventually advance to metastatic disease, where the treatment intent is palliative rather than curative. Metastatic prostate cancer is predominantly treated with androgen deprivation therapy (ADT), which improves overall survival but has been shown to contribute to morbidity. ADT is associated with severe adverse effects that threaten a patient's physical function, such as a loss of muscle mass and bone mineral density, body fat accumulation and fatigue [35]. Consequently, ADT has been linked to impairments across a range of physical fitness qualities, including reductions in muscle mass and strength, walking speed and peak aerobic performance [36, 37]. For men who experience disease progression despite treatment with ADT or are diagnosed with an aggressive form of prostate cancer, the addition of second‐generation androgen receptor inhibitors (ARI) may provide a survival advantage [38]. The use of ARI is, however, associated with greater impairments of physical function and cognition, a higher risk of falls and fractures and worsening of fatigue [39, 40, 41]. The various ways in which treatments such as ADT and ARI interfere with the ability to engage in activities of daily living suggest that PA levels of men with advanced prostate cancer may be lower than the recommended level, but studies that have investigated this are lacking. Trinh et al. [42] assessed PA among men treated with ADT and found that they were highly sedentary; however, only a fraction of the participants had metastatic disease, and no information regarding the use of ARI was provided.

The primary objective of this study was to assess the baseline PA level of men with advanced prostate cancer enrolled in the prospective INTERVAL‐GAP4 trial by comparing their self‐reported weekly moderate‐to‐vigorous PA (MVPA) to the American Cancer Society guidelines. Additionally, we investigated possible associations of MVPA with physical fitness. As a secondary objective, we compared users of second‐generation ARI, such as Apalutamide, Enzalutamide or Darolutamide, to non‐users to assess whether PA and fitness outcomes were worse among men receiving ARI for advanced prostate cancer.

## 2 Methods

### 2.1 Study Design and Participants

Baseline data from participants of the two‐year multicentre INTERVAL‐GAP4 exercise intervention trial was analysed for this study. Participants were recruited globally at 15 INTERVAL‐GAP4 sites: German Sport University, Cologne, Germany; Edith Cowan University, Perth, Australia; Queensland University of Technology, Brisbane, Australia; Victoria University, Melbourne, Australia; University of Alberta, Edmonton, Canada; Cedars Sinai, Los Angeles, CA, United States; Oregon Health and Science University, Portland, OR, United States; University of Colorado, Denver, CO, United States; Fred Hutchinson Cancer Centre, Seattle, WA, United States; University of California San Francisco, San Francisco, CA, United States; Erasmus Medical Centre, Rotterdam, Netherlands; University of Glasgow, Glasgow, United Kingdom; Queens University, Belfast, Ireland; University of Surrey, Guildford, United Kingdom; and King's College, London, United Kingdom.

The INTERVAL‐GAP4 study protocol has been published previously [43] but inclusion criteria were later amended to accommodate changes in clinical practice. Men with histologically documented adenocarcinoma of the prostate and systemic metastatic disease, who had confirmed castrate levels of testosterone (< 50 ng·dL^−1^) due to orchiectomy or treatment with a gonadotropin‐releasing hormone agonist or antagonist, were eligible. In terms of prostate cancer stage at enrolment, men with metastatic hormone‐sensitive prostate cancer (mHSPC), who matched the criteria for high‐risk or high‐volume disease, or metastatic castrate‐resistant prostate cancer (mCRPC) were eligible. Due to the nature of the INTERVAL‐GAP4 exercise intervention trial, men were excluded if they regularly participated in vigorous aerobic exercise for more than 1 h or structured resistance exercise more than once per week. However, there were no restrictions on habitual PA of any intensity level. Data collection included demographic and clinical participant characteristics, PA assessment via a self‐reported questionnaire and exercise testing to assess physical fitness. Eligible participants who completed the baseline measures were randomised into either an exercise intervention or a control group, yet all data presented in this article were collected prior to randomisation. The INTERVAL‐GAP4 trial was prospectively registered (ClinicalTrials.gov: NCT02730338, German Clinical Trials Register: DRKS00010310) and approved by the respective research ethics boards, with the research question of this study retrospectively formulated. Written informed consent was obtained from participants prior to inclusion, and all study procedures were performed in line with the Declaration of Helsinki.

### 2.2 Self‐Reported Physical Activity

PA was assessed using a modified version of the Godin‐Shephard Leisure‐Time Physical Activity Questionnaire (GSLTPAQ) [44], which is considered a standard tool for cancer populations [45]. The self‐administered questionnaire consists of three items to assess the number of times the participant engaged in light, moderate and strenuous PA bouts of at least 15 min duration in the past 7 days. The questionnaire provided examples of aerobic activities for each intensity level. The GSLTPAQ was modified by adding one item to record the frequency and duration of any resistance exercise. Participants at non‐native English‐speaking sites received a translated version of the GSLTPAQ in the local language. Weekly MVPA in minutes was calculated as [MVPA = 2 × (frequency of vigorous PA × duration of vigorous PA) + (frequency of moderate PA × duration of moderate PA)] [25]. Participants who reported at least 150 min of weekly MVPA met the aerobic MVPA guidelines [25, 26]. To facilitate comparison with other studies that used the GSLTPAQ, we also reported the leisure score index (LSI), which is a measure specific to this questionnaire. The LSI is calculated as [LSI = (9 × frequency of vigorous PA) + (5 × frequency of moderate PA) + (3 × frequency of light PA)] [46]. Previous studies have established cut‐points to determine the PA status of participants based on the LSI, with a LSI ≥ 24 considered active and a LSI < 24 considered insufficiently active [47].

### 2.3 Physical Fitness Assessments

Physical fitness was assessed during two separate exercise testing visits performed within 7 days except for the University of California San Francisco, which completed the testing during a single day. The first visit included the cardiopulmonary exercise test (CPET), while 400 m walk time and maximal strength were assessed at the second visit. Participants were told to refrain from exercise in the 48 h prior to each visit. Prior to exercise testing, a standard medical check‐up, including blood pressure measurement, lung function test, resting echocardiogram (ECG) recording and blood draw for a complete blood count, was performed to verify absolute neutrophil and platelet count eligibility criteria. Participants received clearance to perform the CPET by a medical professional.

A medically supervised, symptom‐limited CPET with 12‐lead ECG recording and respiratory gas exchange analysis was performed on a stationary cycle ergometer to assess aerobic fitness. After a 4‐min warm‐up without resistance, participants cycled for 1 min at an initial load of 20 W and each minute thereafter the load increased by either 10 or 15 W, depending on the estimated fitness of the participant. The CPET was stopped when the participant reached volitional exertion, defined as a rating of perceived exertion (RPE) ≥ 9 on the 10‐point Borg scale, or when the cadence dropped below 50 rpm. In addition to subjective exertion, maximal volitional exertion was determined either by a respiratory exchange ratio > 1.10 or a heart rate (HR) within 10 beats of the age‐predicted maximum. Maximal workload (*W*_max_) during the incremental test was recorded as the last completed increment and divided by bodyweight to obtain the relative *W*_max_. Maximal HR (HR_max_) was measured using a chest strap heart rate monitor to verify that participants performed the test to the maximum, which was assumed when the measured HR_max_ was no more than 10 beats per minute below the age‐appropriate HR_max_. Respiratory gas exchange was measured continuously breath by breath during the CPET, and peak oxygen consumption (VO_2_peak) was defined as the highest VO_2_ value.

Functional performance was assessed in a 400 m walk test, which was performed as 10 laps of 40 m. After one warm‐up round, participants were instructed to walk 400 m as fast as possible without running. Time to completion and RPE on the 10‐point Borg scale at the end of the test were recorded. Maximal strength was determined as the one‐repetition maximum (1RM) of leg press, chest press, leg extension and seated row. Based on a review of the most recent imaging results regarding the location and presentation of bone metastases for each participant, an exercise specialist determined whether these exercises were considered safe and those determined to be unsafe were excluded. The warm‐up for each exercise consisted of six repetitions at 60% of the estimated 1RM and three repetitions at 80% of the estimated 1RM with 2 min of rest between sets. The 1RM was then determined as the maximal weight that a participant was able to complete the exercise with using the correct technique. Participants had a maximum of five attempts to reach the 1RM for each exercise with 2 min of rest between attempts. Maximal handgrip strength of the dominant hand was assessed in a seated position using a handgrip dynamometer. Participants were instructed to set their elbow at 90° flexion while keeping a neutral wrist position and rest the lower arm against their upper thigh. Each participant performed three attempts with 30 s of rest between attempts, and the highest value was recorded.

### 2.4 Statistical Analyses

Descriptive statistics were used to present demographic and clinical characteristics, as well as PA (i.e., light PA, moderate PA, vigorous PA, MVPA and resistance exercise) and fitness estimates. Quantile regression analysis was used to examine the associations of physical fitness outcomes, that is, relative VO_2_peak, relative *W*_max_, 400 m walk time, relative 1RM of leg extension, leg press, chest press and seated row and handgrip strength (dependent variables), with MVPA (independent variable) at the 25^th^, 50^th^ and 75^th^ percentiles of the dependent variables. Quantile regression allows the distinction of associations between the independent and dependent variables at different parts of the distribution of the dependent variables while analysing the entire sample, which results in improved statistical power [48]. In contrast to linear regression, the quantile regression coefficient *β* represents the change in the value at each modelled percentile, as opposed to the mean, of the dependent variable. For the subgroup analysis of ARI use, separate quantile regression models were calculated for ARI users and non‐users with the exception of all strength outcomes but leg extension, because the number of participants with complete data was insufficient for a subgroup analysis. All models were adjusted for the same selection of covariates considered to be potential confounders based on previous studies of PA among individuals with cancer [42, 49]. These covariates used for model adjustment included age, body mass index (BMI), time since diagnosis, time on ADT and prostate cancer stage at enrolment (mHSPC or mCRPC). Notably, time on ADT was defined as the time since the current ADT was started and does not include previous treatments with ADT if interrupted before starting the current treatment. Additionally, we performed a between‐group analysis of ARI users and non‐users. Normality was assessed by inspection of graphical representations of the data, such as histograms. Where normality could not be assumed, a Mann‐Whitney‐U test was calculated to determine differences between ARI users and non‐users for continuous variables, with results presented as median (25^th^, 75^th^ percentile). The Pearson's chi‐square test was used to determine between‐group differences for dichotomous variables. Where multiple significance tests were carried out, a Bonferroni correction was applied to adjust the alpha level for the number of tests to reduce the chance of a type 1 error to the 5% level. Participants with missing data for PA or fitness variables were excluded from the analyses with the exception of the strength assessments, which were only completed by a subsample of participants. Therefore, an analysis of this subsample was performed to determine associations of PA with strength outcomes. Missing data for any of the covariates was replaced using a random forest model [50]. A sensitivity analysis for the primary analysis of associations between MVPA and physical fitness variables was performed whereby individual sites were excluded to ascertain if results varied substantially between sites. Statistical significance was defined as *p* < 0.05. All statistical analyses were performed using R (Version 4.3.0, R Core Team, Vienna, Austria) [51] and IBM SPSS Statistics for Windows (Version 29.0, IBM Corp., Armonk, NY, United States).

## 3 Results

### 3.1 Participant Characteristics

**FIGURE 1:**
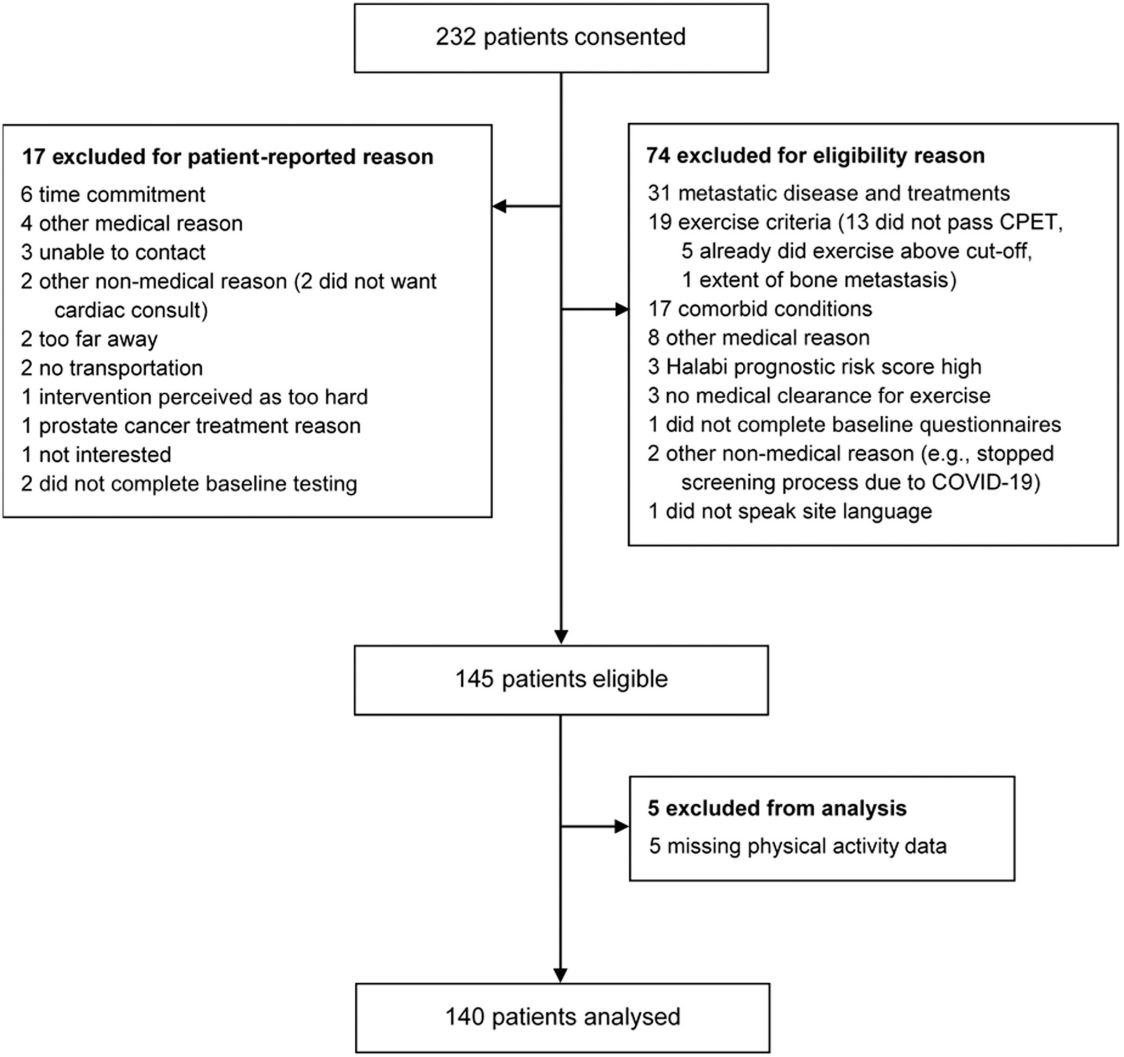
Flow diagram of participants. Excluded patients may have had more than one exclusion criteria noted. This study only included data collected at baseline of INTERVAL‐GAP4. CPET, cardiopulmonary exercise testing.

Between April 2016 and February 2023, a total of 232 men with metastatic prostate cancer were screened for eligibility and 145 were considered eligible for the multicentre INTERVAL‐GAP4 trial. Five participants were excluded due to missing PA data, resulting in 140 participants included in the analysis for this research (Figure 1). Demographic and clinical characteristics of the participants are presented in Table 1. Briefly, participants were on average 69.3 ± 8.4 years of age, had a BMI of 29.2 ± 4.7 m·kg^−2^ and had been diagnosed with prostate cancer for 69 ± 66 months. Thirty‐eight (27%) and 102 (73%) participants had mHSPC and mCRPC at the time of enrolment, respectively. The mean treatment time with ADT was 37 ± 42 months and the mean testosterone serum concentration was 11.4 ± 9.6 ng∙dL^−1^. In addition to ADT, 59 (42%) participants received second‐generation ARI.

**TABLE 1:** Demographic and clinical participant characteristics.

	All participants (*n* = 140)
	Mean (SD) or *n* (%)
**Demographic characteristics**	
Age, years, mean (SD)	69.3 (8.4)
Height, m, mean (SD)	1.75 (0.08)
Weight, kg, mean (SD)	89.2 (16.3)
BMI, m·kg^−2^, mean (SD)	29.2 (4.7)
**Employment status**, *n* (%)	
Retired	96 (69)
Full‐time	19 (14)
Part‐time	16 (11)
Volunteer work	1 (1)
Unemployed	3 (2)
Unable to work	4 (3)
Unknown	1 (1)
**Smoking status**, *n* (%)	
Non‐smoker	60 (43)
Active smoker	11 (8)
Previous smoker	68 (49)
Unknown	1 (< 1)
**Clinical characteristics**	
Time since diagnosis, months, mean (SD)	69 (66)
Time on ADT, months, mean (SD)	37 (42)
Testosterone, ng·dL^−1^, mean (SD)	11.4 (9.6)
PSA, ng mL^−1^, mean (SD)	6.6 (13.9)
**Disease stage**, *n* (%)	
Hormone‐sensitive	38 (27)
Castrate‐resistant	102 (73)
**Metastases**, *n* (%)	
Bones	113 (81)
Lymph nodes	83 (59)
Lung	6 (4)
Liver	4 (3)
Other	5 (4)
**ARI use**, *n* (%)	
Yes	59 (42)
Apalutamide	12 (8)
Enzalutamide	47 (33)
No	81 (58)
**ECOG performance status**, *n* (%)	
0	101 (72)
1	39 (28)
**Bone pain**, *n* (%)	
No pain	110 (79)
Mild pain	30 (21)

Abbreviations: ADT, androgen deprivation therapy; ARI, androgen receptor inhibitor; BMI, body mass index; ECOG, Eastern Cooperative Oncology Group; PSA, prostate‐specific antigen; SD, standard deviation.

### 3.2 Physical Activity and Meeting the Guidelines

Participants self‐reported engaging in light PA, MVPA and resistance exercise for a median of 40 (0, 120), 60 (0, 180) minutes per week and 0 (0, 1) days per week, respectively (Table 2). Sixty‐two (44%) participants self‐reported engaging in no MVPA. Forty‐one (29%) participants met the aerobic PA guidelines for cancer survivors by achieving at least 150 min of MVPA per week. Only twenty‐eight (20%) participants self‐reported engaging in resistance exercise.

**TABLE 2:** Baseline physical activity and physical fitness estimates of all participants.

	All participants (*n* = 140)
	Median (25^th^, 75^th^ percentile) or *n* (%)
**Physical activity (modified GSLTPAQ)**	
Light physical activity, min·week^−1^	40 (0, 120)
Moderate physical activity, min·week^−1^	30 (0, 120)
Vigorous physical activity, min·week^−1^	0 (0, 0)
Moderate‐to‐vigorous physical activity (MVPA), min·week^−1^	60 (0, 180)
Resistance exercise, days·week^−1^	0 (0, 1)
Leisure score index (LSI), a.u.	20 (6, 28)
**Meeting aerobic physical activity guidelines**	
Meeting MVPA guidelines (≥ 150 min·week^−1^)	41 (29)
Not meeting MVPA guidelines (< 150 min·week^−1^)	99 (71)
Meeting LSI guidelines (≥ 24)	46 (33)
Not meeting LSI guidelines (< 24)	94 (67)
**Physical fitness**	
Relative VO_2_peak, mL·min^−1^·kg^−1^	19.7 (16.0, 23.7)
Maximal workload *W*_max_, W	128.0 (100.0, 155.5)
Relative *W*_max_, W·kg^−1^	1.5 (1.1, 1.8)
400 m walk time, s	268.8 (238.8, 309.8)
Relative leg extension 1RM, kg·kg^−1^ ^a^	0.7 (0.5, 0.9)
Relative leg press 1RM, kg·kg^−1^ ^b^	1.2 (1.0, 1.5)
Relative chest press 1RM, kg·kg^−1^ ^c^	0.5 (0.4, 0.6)
Relative seated row 1RM, kg·kg^−1^ ^d^	0.7 (0.6, 0.9)
Handgrip strength, kg^e^	38.7 (32.7, 46.0)

Abbreviations: 1RM, one‐repetition maximum; a.u., arbitrary unit; GSLTPAQ, Godin‐Shephard Leisure‐Time Physical Activity Questionnaire; LSI, leisure score index; VO_2_peak, peak oxygen consumption; *W*_max_, maximal workload.

^a^ Participants with relative leg extension 1RM data: *n* = 115.

^b^ Participants with relative leg press 1RM data: *n* = 49.

^c^ Participants with relative chest press 1RM data: *n* = 51.

^d^ Participants with relative seated row 1RM data: *n* = 48.

^e^ Participants with handgrip strength data: *n* = 57.

### 3.3 Associations of MVPA With Physical Fitness

Adjusted quantile regression estimates of MVPA at the 25^th^, 50^th^ and 75^th^ percentiles of the physical fitness outcomes are presented in Table 3. Each one‐hour increase in weekly MVPA showed a statistically significant association with an increase in relative VO_2_peak at the 25^th^ percentile (*β* = 0.53, *p* = 0.020) and 75^th^ percentile (*β* = 0.66, *p* = 0.001), as well as relative *W*_max_ at the 25^th^ percentile (*β* = 0.05, *p* = 0.003), 50^th^ percentile (*β* = 0.05, *p* = 0.009) and 75^th^ percentile (*β* = 0.07, *p* = 0.004). MVPA was inversely associated with 400 m walk time at the 75^th^ percentile (*β* = −4.26, *p* = 0.023) but not at the 25^th^ or 50^th^ percentiles (*p* > 0.05). There were no statistically significant associations between MVPA and any of the strength outcomes at the 25^th^, 50^th^ or 75^th^ percentiles (*p* > 0.05). Sensitivity analysis of the study site showed that the results did not meaningfully change when individual sites were excluded from the analysis (Table S1).

**TABLE 3:** Adjusted quantile regression estimates of moderate‐to‐vigorous physical activity (MVPA) at the 25^th^, 50^th^ and 75^th^ percentiles of physical fitness outcomes of all participants (*n* = 140), as well as separated into ARI users (*n* = 59) and non‐users (*n* = 81).

Physical activity (hours × week^−1^)^a^	p25	p50	p75
	*β* (95% CI)	*β* (95% CI)	*β* (95% CI)
**MVPA**	**Relative VO**_**2**_ **peak (mL·min** ^**−1**^·**kg** ^**−1**^ **)**		
All participants	0.53 (0.20, 0.84)*	0.42 (0.21, 0.95)	0.66 (0.30, 1.11)***
ARI users	0.51 (−0.25, 0.60)	0.37 (0.20, 1.25)	0.69 (0.20, 1.72)
Non‐users	0.82 (0.20, 1.07)**	0.79 (0.12, 1.03)*	0.46 (0.39, 1.65)
**MVPA**	**Relative *W***_**max**_ **(W·kg** ^**−1**^ **)**		
All participants	0.05 (0.02, 0.08)**	0.05 (0.03, 0.10)**	0.07 (0.03, 0.12)**
ARI users	0.06 (−0.00, 0.12)*	0.07 (0.01, 0.09)	0.05 (0.00, 0.14)
Non‐users	0.05 (0.02, 0.07)	0.05 (0.02, 0.15)	0.09 (0.01, 0.14)*
**MVPA**	**400 m walk time (s)**		
All participants	−2.56 (−9.80, −1.41)	−3.60 (−5.33, −1.02)	−4.26 (−7.20, −1.35)*
ARI users	−2.79 (−10.83, 0.11)	−2.90 (−6.61, 0.18)	−4.06 (−6.65, 0.44)
Non‐users	−8.53 (−10.63, −0.60)**	−4.81 (−11.39, 2.15)	−4.41 (−10.79, 2.65)
**MVPA**	**Relative leg extension 1RM (kg·kg** ^**−1**^ **)** ^b^		
All participants	−0.00 (−0.02, 0.02)	0.01 (−0.01, 0.03)	0.01 (−0.01, 0.02)
ARI users	0.01 (−0.00, 0.03)	0.01 (−0.01, 0.03)	0.02 (0.00, 0.03)
Non‐users	−0.01 (−0.07, 0.02)	0.01 (−0.03, 0.06)	0.01 (−0.02, 0.05)
**MVPA**	**Relative leg press 1RM (kg·kg** ^**−1**^ **)** ^c^		
All participants	0.01 (−0.19, 0.05)	0.01 (−0.06, 0.08)	−0.01 (−0.06, 0.08)
ARI users	NA	NA	NA
Non‐users	NA	NA	NA
**MVPA**	**Relative chest press 1RM (kg·kg** ^**−1**^ **)** ^d^		
All participants	0.01 (−0.05, 0.02)	0.01 (−0.01, 0.03)	0.04 (−0.01, 0.14)
ARI users	NA	NA	NA
Non‐users	NA	NA	NA
**MVPA**	**Relative seated row 1RM (kg·kg** ^**−1**^ **)** ^e^		
All participants	−0.00 (−0.10, 0.02)	−0.01 (−0.02, 0.02)	0.01 (−0.03, 0.06)
ARI users	NA	NA	NA
Non‐users	NA	NA	NA
**MVPA**	**Handgrip strength (kg)** ^f^		
All participants	0.62 (−0.65, 1.80)	0.74 (−0.17, 1.39)	0.89 (−0.42, 2.16)
ARI users	NA	NA	NA
Non‐users	NA	NA	NA

*Note:* All models were adjusted for age, body mass index, prostate cancer stage, time since diagnosis and time on androgen deprivation therapy.

Abbreviations: 1RM, one‐repetition maximum; *β*, unstandardised regression coefficient; ARI, androgen receptor inhibitor; CI, confidence interval; MVPA, moderate‐to‐vigorous physical activity; NA, not available; VO_2_peak, peak oxygen consumption; *W*_max_, maximal workload.

^a^ MVPA was analysed in hours week^−1^ to provide more interpretable *β* coefficients.

^b^ Participants with relative leg extension 1RM data: all participants, *n* = 115; ARI users, *n* = 44; non‐users, *n* = 71.

^c^ Participants with relative leg press 1RM data: all participants, *n* = 49; ARI users, *n* = 18; non‐users, *n* = 31.

^d^ Participants with relative chest press 1RM data: all participants, *n* = 51; ARI users, *n* = 21; non‐users, *n* = 30.

^e^ Participants with relative seated row 1RM data: all participants, *n* = 48; ARI users, *n* = 21; non‐users, *n* = 27.

^f^ Participants with handgrip strength data: all participants, *n* = 57; ARI users, *n* = 24, non‐users, *n* = 33.

* *p* ≤ 0.05.

** *p* ≤ 0.01.

*** *p* ≤ 0.001.

### 3.4 MVPA and Physical Fitness According to ARI Use

There were no statistically significant differences between ARI users and non‐users for any of the PA or physical fitness variables at the *p* > 0.003 alpha level, which was adjusted using Bonferroni correction (Table 4). In ARI users, each one‐hour increase in weekly MVPA showed a statistically significant association with an increase in relative *W*_max_ at the 25^th^ percentile (*β* = 0.06, *p* = 0.046) but not at the 50^th^ or 75^th^ percentiles, even though the direction and strength of the associations were similar (*p* > 0.05) (Table 3). ARI users showed no statistically significant associations between MVPA and relative VO_2_peak or 400 m walk time (*p* > 0.05). In non‐users, each 1‐h increase in weekly MVPA showed a statistically significant association with an increase in relative VO_2_peak at the 25^th^ percentile (*β* = 0.82, *p* = 0.008) and 50^th^ percentile (*β* = 0.79, *p* = 0.014) but not at the 75^th^ percentile (*p* > 0.05). Non‐users also showed a statistically significant association of MVPA with relative *W*_max_ at the 75^th^ percentile (*β* = 0.09, *p* = 0.041) and 400 m walk time at the 25^th^ percentile (*β* = −8.53, *p* = 0.010). Neither group showed a statistically significant association of MVPA with relative leg extension 1RM at the 25^th^, 50^th^ or 75^th^ percentiles (*p* > 0.05).

**TABLE 4:** Participant characteristics, physical activity and physical fitness of ARI users and non‐users.

	ARI users (*n* = 59)	Non‐users (*n* = 81)	*p* ^f^
	Median (25^th^, 75^th^ percentile) or *n* (%)	Median (25^th^, 75^th^ percentile) or *n* (%)	
**Participant characteristics**			
Age, years	71.0 (64.0, 73.5)	69.0 (64.0, 75.0)	0.710
BMI, m·kg^−2^	28.7 (25.6, 30.9)	28.9 (25.9, 32.8)	0.272
Time since diagnosis, months	56 (19, 106)	36 (18, 95)	0.386
Time on ADT, months	19 (6, 51)	21 (10, 44)	0.884
**Physical activity**			
Moderate physical activity, min·week^−1^	60 (0, 173)	0 (0, 90)	0.012
Vigorous physical activity, min·week^−1^	0 (0, 20)	0 (0, 0)	0.558
Moderate‐to‐vigorous physical activity (MVPA), min·week^−1^	120 (0, 273)	0 (0, 120)	0.012
Meeting MVPA guidelines			0.011
Yes (MVPA ≥ 150·min week^−1^)	24 (41)	17 (21)	
No (MVPA < 150·min week^−1^)	35 (59)	64 (79)	
**Physical fitness**			
Relative VO_2_peak, mL·min^−1^·kg^−1^	20.0 (16.3, 22.9)	19.5 (15.5, 24.9)	0.841
Relative *W*_max_, W·kg^−1^	1.5 (1.1, 1.9)	1.5 (1.1, 1.7)	0.469
400 m walk time, s	270.4 (238.5, 308.5)	268.0 (239.1, 314.9)	0.854
Relative leg extension 1RM, kg·kg^−1^ ^a^	0.7 (0.5, 0.9)	0.7 (0.5, 0.9)	0.950
Relative leg press 1RM, kg·kg^−1^ ^b^	1.2 (1.0, 1.5)	1.2 (0.9, 1.4)	0.494
Relative chest press 1RM, kg·kg^−1^ ^c^	0.5 (0.4, 0.6)	0.5 (0.4, 0.6)	0.151
Relative seated row 1RM, kg·kg^−1^ ^d^	0.7 (0.6, 0.8)	0.8 (0.6, 0.9)	0.194
Handgrip strength, kg^e^	38.5 (33.5, 46.5)	38.7 (32.7, 42.4)	0.728

Abbreviations: 1RM, one‐repetition maximum; ADT, androgen deprivation therapy; ARI, androgen receptor inhibitor; BMI, body mass index; MVPA, moderate‐to‐vigorous physical activity; VO_2_peak, peak oxygen consumption; *W*_max_, maximal workload.

^a^ Participants with relative leg extension 1RM data: all participants, *n* = 115; ARI users, *n* = 44; non‐users, *n* = 71.

^b^ Participants with relative leg press 1RM data: all participants, *n* = 49; ARI users, *n* = 18; non‐users, *n* = 31.

^c^ Participants with relative chest press 1RM data: all participants, *n* = 51; ARI users, *n* = 21; non‐users, *n* = 30.

^d^ Participants with relative seated row 1RM data: all participants, *n* = 48; ARI users, *n* = 21; non‐users, *n* = 27.

^e^ Participants with handgrip strength data: all participants, *n* = 57; ARI users, *n* = 24, non‐users, *n* = 33.

^f^ Bonferroni correction was applied to adjust the alpha level for the number of tests to *α* = 0.003.

## 4 Discussion

In advanced prostate cancer, the ability to engage in PA is complicated by the use of treatments such as ADT and ARI that inhibit testosterone signalling. Therefore, we assessed the PA of men with advanced prostate cancer and determined if they met PA guidelines in a sample of men who were required to meet PA cut‐offs of a maximum of 1 h of vigorous aerobic exercise and one structured resistance exercise session per week. As expected, we found that overall levels of MVPA were driven mostly by moderate intensity PA, with participants reporting an average of 128 min MVPA per week. Other randomised controlled trials (RCT) that assessed PA at baseline using the GSLTPAQ reported higher PA levels when there was no pre‐set exclusion criterion for maximal weekly PA. In a sample of breast, prostate and colorectal cancer survivors, participants reported an average of 202 min MVPA per week [30]. Their average age was, however, 5 years younger than our sample, and most of the participants were female. Contrary to this observation of higher PA levels, Papadopoulos et al. reported only 68 min of weekly MVPA among men with prostate cancer on ADT participating in an RCT, which excluded men engaging in more than 150 min of weekly MVPA [52]. Importantly, statistical analysis showed a non‐normal distribution of MVPA in the present study as indicated by a median of 60 (25^th^ percentile, 75^th^ percentile: 0, 180) min of MVPA per week, likely due to the requirement that participants be less active, which would have truncated the distribution at the upper end.

MVPA as a widely used measure of PA provides an advantage over the original measure proposed by the GSLTPAQ, the LSI, because it facilitates comparison with studies that assessed PA using different methods. Calculating MVPA based on the GSLTPAQ estimates of moderate and vigorous PA also allowed us to determine whether participants met the PA guidelines set out by the American Cancer Society. In our study, only 29% of participants achieved the recommended level of MVPA. The primary aim of the INTERVAL‐GAP4 trial was to assess whether a two‐year intervention focused on supervised high‐intensity aerobic and resistance training compared to self‐directed exercise improved overall survival. Therefore, we intentionally enrolled men who were less active and only engaged in limited vigorous structured aerobic and resistance exercise, whereas habitual PA was not restricted. Despite these eligibility criteria of the INTERVAL‐GAP4 trial, our findings are in line with results from other studies in individuals with lymphoma [27], breast cancer [53] and localised prostate cancer [54], which did not specify excluding participants above a certain PA threshold. A markedly higher level of PA was reported by Santa Mina et al. [55] in men with prostate cancer prior to prostatectomy, who found that 46% met the guidelines, although their participants were at an early disease stage and on average 9 years younger than our sample. To the contrary, Ozdemir et al. [56] found that only 21% of newly diagnosed prostate cancer patients were sufficiently active despite a lower mean age and BMI than our sample. Both of these were observational studies and did not report excluding participants based on their PA or exercise levels. Our data suggest that restricting eligibility of men with metastatic prostate cancer based on vigorous aerobic and strength exercise will still result in a fairly active sample in terms of moderate intensity PA. However, caution should be applied when analysing PA guideline compliance based on self‐reported PA. A study of men with localised prostate cancer found that an astounding 73% of participants met the PA guidelines according to self‐reported MVPA, though only 11% met the PA guidelines when using accelerometer‐derived MVPA [29].

Reporting bias and false classification of PA intensities are recognised issues when using self‐reported PA estimates, especially in older populations with potentially lower cognitive function [57]. To illustrate this point, Sloane et al. compared self‐reported to accelerometer‐derived PA in prostate and breast cancer survivors [58]. They observed a marked discrepancy of absolute values, with participants accumulating 59 min of self‐reported versus 228 min of accelerometer‐derived MVPA per week, although there was a significant positive correlation between both measures. Similarly, Papadopoulos et al. [52] found that accelerometer‐derived data showed an additional 80 min of time spent in MVPA compared to self‐reported data. The discrepancy between absolute values of self‐reported and device‐measured PA raises the question of which method should be implemented when determining whether individuals meet PA guidelines.

Physical inactivity is generally associated with poorer outcomes across several health domains [59, 60, 61] and, in return, poor health can limit physical activity engagement in older adults [62]. Many studies have reported a link between overall health status and physical fitness, which is why the latter has been recognised as a powerful marker of health [1, 5]. To investigate a potential link between PA and physical fitness, we analysed their association using quantile regression analysis. We found that higher weekly MVPA was associated with a statistically significant improvement in relative VO_2_peak and *W*_max_ across most percentiles, despite restricting vigorous aerobic exercise to a maximum of 1 h per week. Unstandardised beta coefficients from our adjusted models showed that for every 1‐h increase in weekly MVPA, relative VO_2_peak increases by 0.53 mL·min^−1^·kg^−1^ at the 25^th^ percentile and 0.66 mL·min^−1^·kg^−1^ at the 75^th^ percentile. A minimal clinically important difference of 2.5 mL·min^−1^·kg^−1^ for VO_2_peak improvements has been suggested previously [63], which would require 33–40 min of additional MVPA per day according to our findings. Our results are consistent with previous studies, which found that less active prostate cancer survivors had a lower VO_2_peak and that greater cumulative MVPA during breast cancer treatment was related to higher post‐treatment cardiorespiratory fitness [64, 65].

Positive associations between a higher MVPA and faster walking speed have been reported in women with ovarian cancer [66]. Furthermore, Demark‐Wahnefried et al. [67] observed that older cancer survivors who exercised regularly had significantly higher physical function scores. These studies support our findings of an inverse association between MVPA and 400 m walk time. However, in our study, this benefit of PA was only present for men in the highest percentile of walk time, that is, the slowest walking speed, which suggests a potential plateau of the benefit of habitual PA. On the other hand, we did not observe an association between higher weekly MVPA and improved strength. These results are in line with a previous study in head and neck cancer survivors [28], which found that active participants did not differ in their grip strength from inactive participants, whereas a significant association between higher MVPA and better handgrip strength was found in colorectal cancer survivors [68]. The lack of a statistically significant association of PA with strength outcomes in our study is likely related to the fact that the GSLTPAQ specifically captures aerobic physical activity and that not all participants were able to complete the strength assessments due to bone metastasis, so the analysis may have been underpowered. Furthermore, men who performed regular structured resistance training were excluded, and even though one weekly resistance exercise session was allowed, only 20% of participants reported regularly engaging in resistance exercise. Androgen deprivation is known to have detrimental effects on muscles, such as loss of muscle mass and strength [35]. In fact, while supervised exercise has been shown to improve muscle strength in men on ADT, study results of its effects on muscle mass are inconsistent [69]. Enhancing neuromuscular fitness during ADT likely requires a targeted stimulus such as resistance training. Because it has been reported that the rate of ADT‐related muscle mass loss may be linked to the treatment time [70], we adjusted all models for the length of ADT treatment. Furthermore, because PA appears to decrease with older age [71], we also adjusted all models for age.

Additionally, we performed a subgroup analysis to investigate whether MVPA and fitness outcomes differed in men receiving ARI in addition to ADT. ARI were originally approved for advanced prostate cancer but are more and more frequently utilised at earlier disease stages [72]. Despite the increase in ARI use, data on its toxicities for neuromuscular and cardiorespiratory health are lacking. We found that the positive association between MVPA and relative VO_2_peak only persisted in non‐users but not ARI users. Similarly, higher MVPA was only linked to a faster walk time in non‐users. When comparing both groups, we observed no statistically significant differences in PA behaviour and physical fitness. While a large meta‐analysis demonstrated that ARI use may impair lower limb muscle function, which results in a significantly increased risk of falls and fractures [39], this cannot be concluded from our study. However, our findings suggest that ARI, which directly interferes with the androgen receptor and elicits an even stronger androgen blockade than ADT alone, might blunt the benefits of PA for cardiorespiratory and walking fitness. Given the increasing use of ARI and the expansion to earlier prostate cancer stages, which makes these drugs available to men of various age groups and levels of physical function, more studies are needed to elucidate the effects of ARI on physical fitness and activity.

### 4.1 Strengths and Limitations

Our study has several notable strengths. The INTERVAL‐GAP4 trial is a large, multinational study of men with advanced prostate cancer on ADT. Due to the specific physiological changes and resulting side effects associated with ADT, treatment homogeneity is critical for studies involving men with prostate cancer. Furthermore, physical fitness was assessed using gold standard methods, that is, respiratory gas exchange and 1RM tests. The additional analysis of PA and fitness data according to ARI use provides valuable and novel information. Another strength is the use of quantile regression analysis, which allowed us to examine the association of MVPA as a PA estimate with different levels of physical fitness without compromising the statistical power.

A major limitation of our study is the cross‐sectional design, which does not allow us to infer causation. Secondly, participants were recruited for the INTERVAL‐GAP4 exercise intervention trial, which applied specific inclusion criteria such as medical clearance to exercise at high intensities. Participants were also excluded if they performed more than 1 h of vigorous aerobic exercise or more than one structured resistance exercise session per week or participated in structured exercise programmes. Thus, the generalisability of our results to men with advanced prostate cancer not meeting these criteria may be limited. Furthermore, recall and social desirability bias are known to affect self‐reported PA data. The self‐reported PA estimates may suggest that some participants engaged in more PA than permitted by the inclusion criteria, which is likely a result of overreporting on the survey. While the inclusion criteria were checked in conversation with each participant that allowed follow‐up questions, the GSLTPAQ was completed independently by the participants. We also suspected older individuals with cancer to spend a large share of their time performing light PA, yet 42% of participants reported not engaging in any light PA. Capturing such activities may require objective measurement methods. For this reason, we cannot provide reliable estimates of light PA, which might be of interest to better understand the PA patterns of men with advanced prostate cancer. In addition, there is a lack of consensus regarding the classification of resistance exercise, which can be argued to qualify as vigorous PA; however, in our study only aerobic activities were assessed by the GSLTPAQ. Lastly, the comparison of ARI users and non‐users is limited by the difference in sample size between groups, which may have resulted in lower statistical power.

## 5 Conclusion

PA as a health‐promoting behaviour and physical fitness as a marker of health are inevitably intertwined. Both are linked to better health outcomes in healthy individuals but become even more relevant in chronic diseases, such as cancer, where the relationship is influenced by physiological changes caused by the disease and its treatments. In our study of men with advanced prostate cancer on ADT, we intentionally enrolled a population with overall low participation in vigorous and structured resistance exercise where the majority of participants failed to meet PA guidelines. Despite these restrictions, higher MVPA was significantly associated with improved cardiorespiratory fitness and walking performance, as expected, but not muscle strength, which is a likely result of men with prostate cancer engaging in aerobic PA rather than resistance exercise. Our findings suggest that in a population of men on ADT that is predominantly inactive according to established standards, clinically meaningful improvements of physical fitness may be achieved by increasing habitual PA, although improving neuromuscular fitness seems to require a more specific stimulus. Consideration of secondary cancer treatments might also be important given that the relationship between PA and fitness outcomes was less pronounced in ARI users than non‐users. These results may be used to design interventions that aim to incorporate PA and, more importantly, targeted exercise medicine as a supportive therapy programme for men with advanced prostate cancer with consideration of their cancer treatment, health and fitness status.

## Author Contributions

**Lisa Umlauff:** conceptualization (equal), formal analysis (lead), investigation (equal), writing – original draft (lead). **Stacey A. Kenfield:** data curation (lead), project administration (equal), writing – review and editing (supporting). **Robert U. Newton:** project administration (equal), writing – review and editing (supporting). **Nicolas H. Hart:** project administration (equal), writing – review and editing (supporting). **Fred Saad:** project administration (equal), writing – review and editing (supporting). **Kerry S. Courneya:** project administration (equal), writing – review and editing (supporting). **Rosemary Greenwood:** project administration (equal), writing – review and editing (equal). **Wilhelm Bloch:** funding acquisition (supporting), supervision (supporting). **INTERVAL‐GAP4 Steering Committee/Coordinating Centres Members/Protocol Development Working Group Members:** funding acquisition (lead), investigation (equal), project administration (lead). **Moritz Schumann:** conceptualization (equal), supervision (lead), writing – review and editing (supporting).

## Ethics Statement

This study was approved by the respective research ethics boards of the participating institutions.

## Consent

All participants provided written informed consent prior to participation.

## Conflicts of Interest

The authors declare no conflicts of interest.

## Supporting information


Table S1.

## Data Availability

Data will be provided upon reasonable request.
