# Supplementary material for: Meeting Aerobic Physical Activity Guidelines and Associations With Physical Fitness in Men With Metastatic Prostate Cancer: Baseline Results of the Multicentre INTERVAL‐GAP4 Trial
Source: Cancer Med. 2024 Dec 4;13(23):e70261. doi: 10.1002/cam4.70261 (PMC11617593; doi:10.1002/cam4.70261)
Supplement: Supplementary file 1 — Table S1. [file CAM4-13-e70261-s001.docx]

**Supplemental Table 1.** Sensitivity analysis for adjusted quantile regression estimates of moderate-to-vigorous physical activity (MVPA) at the 25^th^, 50^th^ and 75^th^ percentiles of the dependent variables, i.e. physical fitness outcomes, of all participants (n = 140).

| **Excluded site (ID)** | **Dependent variable** | **Percentile** | **β** | **95% CI lower limit** | **95% CI upper limit** | **p-value** |
| --- | --- | --- | --- | --- | --- | --- |
| 1 | Relative VO_2_peak | p25 | 0.59817 | 0.0498 | 1.10272 | 0.046 |
|  | Relative VO_2_peak | p50 | 0.7481 | 0.14532 | 1.2798 | 0.016 |
|  | Relative VO_2_peak | p75 | 0.76792 | 0.46342 | 1.74238 | 0.013 |
|  | Relative W_max_ | p25 | 0.04829 | 0.02475 | 0.0791 | 0.040 |
|  | Relative W_max_ | p50 | 0.04957 | 0.01655 | 0.10601 | 0.071 |
|  | Relative W_max_ | p75 | 0.08004 | 0.0345 | 0.16077 | 0.009 |
|  | 400 m walking time | p25 | -3.1701 | -9.52967 | -0.40137 | 0.206 |
|  | 400 m walking time | p50 | -3.64595 | -5.18504 | -0.50689 | 0.189 |
|  | 400 m walking time | p75 | -5.90395 | -7.42543 | -1.65785 | 0.012 |
|  | Relative leg extension 1RM | p25 | -0.01334 | -0.04818 | 0.01607 | 0.372 |
|  | Relative leg extension 1RM | p50 | 1.00E-05 | -0.02147 | 0.0159 | 0.999 |
|  | Relative leg extension 1RM | p75 | 0.00145 | -0.02766 | 0.04233 | 0.933 |
|  | Handgrip strength | p25 | 0.73152 | 0.07154 | 1.1894 | 0.216 |
|  | Handgrip strength | p50 | 0.63839 | -0.26043 | 1.28812 | 0.330 |
|  | Handgrip strength | p75 | 0.76695 | -0.66632 | 2.75401 | 0.351 |
|  | Relative leg press 1RM | p25 | -0.0364 | -0.23027 | 0.05602 | 0.484 |
|  | Relative leg press 1RM | p50 | 0.00643 | -0.05916 | 0.07296 | 0.922 |
|  | Relative leg press 1RM | p75 | 0.0064 | -0.09459 | 0.23704 | 0.946 |
|  | Relative chest press 1RM | p25 | 0.01279 | -0.03598 | 0.02516 | 0.510 |
|  | Relative chest press 1RM | p50 | 0.0105 | -0.00942 | 0.04896 | 0.552 |
|  | Relative chest press 1RM | p75 | 0.03248 | -0.01647 | 0.15991 | 0.265 |
|  | Relative seated row 1RM | p25 | -0.02345 | -0.07078 | 0.00294 | 0.480 |
|  | Relative seated row 1RM | p50 | -0.02097 | -0.03957 | 0.0232 | 0.321 |
|  | Relative seated row 1RM | p75 | -0.03995 | -0.05097 | 0.0604 | 0.127 |
| 2 | Relative VO_2_peak | p25 | 0.52228 | 0.23923 | 0.85816 | 0.022 |
|  | Relative VO_2_peak | p50 | 0.40445 | 0.22189 | 0.93338 | 0.063 |
|  | Relative VO_2_peak | p75 | 0.67031 | 0.40935 | 1.11414 | 0.001 |
|  | Relative W_max_ | p25 | 0.05261 | 0.02132 | 0.08339 | 0.003 |
|  | Relative W_max_ | p50 | 0.05076 | 0.03426 | 0.09673 | 0.012 |
|  | Relative W_max_ | p75 | 0.0704 | 0.03021 | 0.12129 | 0.003 |
|  | 400 m walking time | p25 | -4.29499 | -9.31831 | -1.35238 | 0.025 |
|  | 400 m walking time | p50 | -3.92847 | -5.05113 | -1.01506 | 0.054 |
|  | 400 m walking time | p75 | -5.33829 | -7.80214 | -0.35544 | 0.015 |
|  | Relative leg extension 1RM | p25 | 0.00554 | -0.02421 | 0.03107 | 0.689 |
|  | Relative leg extension 1RM | p50 | 0.0135 | -0.01198 | 0.03309 | 0.350 |
|  | Relative leg extension 1RM | p75 | 0.01376 | -0.01019 | 0.02285 | 0.298 |
|  | Handgrip strength | p25 | 0.73134 | -0.69654 | 1.8961 | 0.292 |
|  | Handgrip strength | p50 | 0.80547 | -0.26097 | 1.70366 | 0.183 |
|  | Handgrip strength | p75 | 0.89056 | 0.34396 | 1.98514 | 0.166 |
|  | Relative leg press 1RM | p25 | 0.00864 | -0.25654 | 0.07017 | 0.812 |
|  | Relative leg press 1RM | p50 | 0.01368 | -0.05572 | 0.13302 | 0.792 |
|  | Relative leg press 1RM | p75 | -0.01053 | -0.08161 | 0.27727 | 0.822 |
|  | Relative chest press 1RM | p25 | 0.01232 | -0.04089 | 0.0192 | 0.234 |
|  | Relative chest press 1RM | p50 | 0.0054 | -0.01141 | 0.03185 | 0.680 |
|  | Relative chest press 1RM | p75 | 0.00905 | -0.01122 | 0.15929 | 0.600 |
|  | Relative seated row 1RM | p25 | -0.00446 | -0.11338 | 0.0227 | 0.788 |
|  | Relative seated row 1RM | p50 | 0.00059 | -0.02142 | 0.03006 | 0.976 |
|  | Relative seated row 1RM | p75 | 0.00645 | -0.02919 | 0.05884 | 0.746 |
| 3 | Relative VO_2_peak | p25 | 0.53142 | 0.1874 | 0.79813 | 0.021 |
|  | Relative VO_2_peak | p50 | 0.42194 | 0.21494 | 0.93588 | 0.061 |
|  | Relative VO_2_peak | p75 | 0.65555 | 0.33662 | 1.10445 | 0.001 |
|  | Relative W_max_ | p25 | 0.05333 | 0.01861 | 0.08287 | 0.002 |
|  | Relative W_max_ | p50 | 0.05192 | 0.03263 | 0.09558 | 0.009 |
|  | Relative W_max_ | p75 | 0.07014 | 0.02923 | 0.12008 | 0.004 |
|  | 400 m walking time | p25 | -2.45853 | -9.74572 | -1.43768 | 0.144 |
|  | 400 m walking time | p50 | -3.64111 | -5.21419 | -0.86264 | 0.066 |
|  | 400 m walking time | p75 | -3.77475 | -7.12463 | -1.97499 | 0.035 |
|  | Relative leg extension 1RM | p25 | 0.0015 | -0.01526 | 0.01753 | 0.903 |
|  | Relative leg extension 1RM | p50 | 0.01188 | -0.01041 | 0.02975 | 0.350 |
|  | Relative leg extension 1RM | p75 | 0.01247 | -0.00661 | 0.02319 | 0.319 |
|  | Handgrip strength | p25 | 0.62224 | -0.64625 | 1.80196 | 0.312 |
|  | Handgrip strength | p50 | 0.74459 | -0.17162 | 1.38979 | 0.236 |
|  | Handgrip strength | p75 | 0.89056 | -0.42254 | 2.15913 | 0.198 |
|  | Relative leg press 1RM | p25 | 0.00512 | -0.21178 | 0.04686 | 0.872 |
|  | Relative leg press 1RM | p50 | 0.01448 | -0.0611 | 0.07547 | 0.746 |
|  | Relative leg press 1RM | p75 | -0.01135 | -0.06762 | 0.07966 | 0.784 |
|  | Relative chest press 1RM | p25 | 0.01203 | -0.04531 | 0.0193 | 0.258 |
|  | Relative chest press 1RM | p50 | 0.00862 | -0.01047 | 0.03229 | 0.532 |
|  | Relative chest press 1RM | p75 | 0.03526 | -0.00505 | 0.14494 | 0.271 |
|  | Relative seated row 1RM | p25 | -0.00307 | -0.099 | 0.01751 | 0.845 |
|  | Relative seated row 1RM | p50 | -0.00256 | -0.01835 | 0.03264 | 0.886 |
|  | Relative seated row 1RM | p75 | 0.00593 | -0.02482 | 0.04944 | 0.742 |
| 4 | Relative VO_2_peak | p25 | 0.41777 | -0.16016 | 0.87471 | 0.107 |
|  | Relative VO_2_peak | p50 | 0.32287 | 0.14579 | 0.87922 | 0.213 |
|  | Relative VO_2_peak | p75 | 0.644 | 0.15665 | 0.8887 | 0.005 |
|  | Relative W_max_ | p25 | 0.03788 | -0.02369 | 0.07007 | 0.063 |
|  | Relative W_max_ | p50 | 0.04743 | 0.02355 | 0.05577 | 0.025 |
|  | Relative W_max_ | p75 | 0.03237 | 0.02128 | 0.0839 | 0.073 |
|  | 400 m walking time | p25 | -1.82876 | -10.01695 | -0.41397 | 0.438 |
|  | 400 m walking time | p50 | -2.21623 | -5.7666 | 0.40296 | 0.422 |
|  | 400 m walking time | p75 | -3.81602 | -6.63201 | 1.55626 | 0.145 |
|  | Relative leg extension 1RM | p25 | -0.00948 | -0.04785 | 0.02195 | 0.553 |
|  | Relative leg extension 1RM | p50 | 0.00304 | -0.02365 | 0.02441 | 0.859 |
|  | Relative leg extension 1RM | p75 | 0.0066 | -0.02303 | 0.02737 | 0.721 |
|  | Handgrip strength | p25 | 0.81276 | -3.62051 | 1.21707 | 0.495 |
|  | Handgrip strength | p50 | 0.72317 | -2.39171 | 2.24146 | 0.580 |
|  | Handgrip strength | p75 | 0.02431 | -2.90916 | 3.13285 | 0.990 |
|  | Relative leg press 1RM | p25 | -0.0275 | -Inf | 0.05396 | 0.475 |
|  | Relative leg press 1RM | p50 | 0.01079 | -0.06126 | 0.03938 | 0.822 |
|  | Relative leg press 1RM | p75 | -0.01386 | -0.07696 | Inf | 0.763 |
|  | Relative chest press 1RM | p25 | 0.01206 | -0.08541 | 0.01977 | 0.320 |
|  | Relative chest press 1RM | p50 | 0.00372 | -0.0065 | 0.06289 | 0.791 |
|  | Relative chest press 1RM | p75 | 0.03623 | -0.00464 | 0.15843 | 0.304 |
|  | Relative seated row 1RM | p25 | -0.00242 | -0.19282 | 0.01809 | 0.885 |
|  | Relative seated row 1RM | p50 | -0.00121 | -0.01994 | 0.03297 | 0.954 |
|  | Relative seated row 1RM | p75 | 0.00589 | -0.03096 | 0.31842 | 0.797 |
| 5 | Relative VO_2_peak | p25 | 0.54295 | 0.12108 | 0.95626 | 0.029 |
|  | Relative VO_2_peak | p50 | 0.48091 | 0.33224 | 0.89161 | 0.034 |
|  | Relative VO_2_peak | p75 | 0.66039 | 0.41008 | 1.17918 | 0.001 |
|  | Relative W_max_ | p25 | 0.05526 | 0.02157 | 0.08856 | 0.002 |
|  | Relative W_max_ | p50 | 0.05408 | 0.03222 | 0.09286 | 0.009 |
|  | Relative W_max_ | p75 | 0.0626 | 0.02604 | 0.11483 | 0.006 |
|  | 400 m walking time | p25 | -4.22258 | -9.81307 | -1.36756 | 0.039 |
|  | 400 m walking time | p50 | -4.36101 | -5.69213 | -1.24655 | 0.055 |
|  | 400 m walking time | p75 | -4.9937 | -7.50709 | -3.78308 | 0.015 |
|  | Relative leg extension 1RM | p25 | -0.00398 | -0.02433 | 0.01715 | 0.769 |
|  | Relative leg extension 1RM | p50 | 0.00989 | -0.01226 | 0.03459 | 0.522 |
|  | Relative leg extension 1RM | p75 | 0.00935 | -0.01512 | 0.02184 | 0.522 |
|  | Handgrip strength | p25 | 0.72584 | -0.47651 | 1.90027 | 0.320 |
|  | Handgrip strength | p50 | 0.68711 | -0.11515 | 1.18218 | 0.334 |
|  | Handgrip strength | p75 | 0.74847 | -0.47361 | 2.1251 | 0.300 |
|  | Relative leg press 1RM | p25 | 0.00908 | -0.37625 | 0.05477 | 0.802 |
|  | Relative leg press 1RM | p50 | 0.01397 | -0.06054 | 0.1078 | 0.784 |
|  | Relative leg press 1RM | p75 | -0.01965 | -0.08246 | 0.21214 | 0.681 |
|  | Relative chest press 1RM | p25 | 0.01203 | -0.04413 | 0.0202 | 0.224 |
|  | Relative chest press 1RM | p50 | 0.00859 | -0.00936 | 0.02889 | 0.468 |
|  | Relative chest press 1RM | p75 | 0.01346 | -0.00833 | 0.10756 | 0.414 |
|  | Relative seated row 1RM | p25 | -0.00676 | -0.11772 | 0.02376 | 0.725 |
|  | Relative seated row 1RM | p50 | -0.0024 | -0.01893 | 0.02575 | 0.906 |
|  | Relative seated row 1RM | p75 | 0.0068 | -0.0308 | 0.04937 | 0.746 |
| 6 | Relative VO_2_peak | p25 | 0.53151 | 0.19681 | 0.89946 | 0.022 |
|  | Relative VO_2_peak | p50 | 0.47787 | 0.22601 | 0.95587 | 0.037 |
|  | Relative VO_2_peak | p75 | 0.65555 | 0.34502 | 1.11519 | 0.001 |
|  | Relative W_max_ | p25 | 0.0566 | 0.02081 | 0.08304 | 0.001 |
|  | Relative W_max_ | p50 | 0.05268 | 0.03433 | 0.09563 | 0.009 |
|  | Relative W_max_ | p75 | 0.07014 | 0.02932 | 0.12019 | 0.004 |
|  | 400 m walking time | p25 | -2.61941 | -9.81966 | -1.5662 | 0.123 |
|  | 400 m walking time | p50 | -3.79586 | -5.31771 | -1.01221 | 0.052 |
|  | 400 m walking time | p75 | -4.29868 | -7.14381 | -0.90174 | 0.025 |
|  | Relative leg extension 1RM | p25 | -0.00019 | -0.01547 | 0.02268 | 0.987 |
|  | Relative leg extension 1RM | p50 | 0.01174 | -0.01161 | 0.02899 | 0.355 |
|  | Relative leg extension 1RM | p75 | 0.01405 | -0.00664 | 0.02272 | 0.276 |
|  | Handgrip strength | p25 | 0.62224 | -0.64625 | 1.80196 | 0.312 |
|  | Handgrip strength | p50 | 0.74459 | -0.17162 | 1.38979 | 0.236 |
|  | Handgrip strength | p75 | 0.89056 | -0.42254 | 2.15913 | 0.198 |
|  | Relative leg press 1RM | p25 | 0.00864 | -0.19384 | 0.04548 | 0.785 |
|  | Relative leg press 1RM | p50 | 0.01448 | -0.06082 | 0.07899 | 0.743 |
|  | Relative leg press 1RM | p75 | -0.01216 | -0.06488 | 0.07628 | 0.742 |
|  | Relative chest press 1RM | p25 | 0.01203 | -0.04741 | 0.01941 | 0.276 |
|  | Relative chest press 1RM | p50 | 0.00862 | -0.01176 | 0.04815 | 0.540 |
|  | Relative chest press 1RM | p75 | 0.03526 | -0.00853 | 0.14731 | 0.281 |
|  | Relative seated row 1RM | p25 | -0.00446 | -0.04434 | 0.02601 | 0.761 |
|  | Relative seated row 1RM | p50 | -0.00567 | -0.01844 | 0.04234 | 0.747 |
|  | Relative seated row 1RM | p75 | 0.00645 | -0.02938 | 0.05609 | 0.718 |
| 7 | Relative VO_2_peak | p25 | 0.55509 | 0.21597 | 0.86987 | 0.024 |
|  | Relative VO_2_peak | p50 | 0.44496 | 0.23142 | 1.10004 | 0.058 |
|  | Relative VO_2_peak | p75 | 0.61999 | 0.31374 | 1.16616 | 0.003 |
|  | Relative W_max_ | p25 | 0.05459 | 0.02136 | 0.08393 | 0.002 |
|  | Relative W_max_ | p50 | 0.05015 | 0.03675 | 0.08554 | 0.012 |
|  | Relative W_max_ | p75 | 0.07123 | 0.02344 | 0.12193 | 0.005 |
|  | 400 m walking time | p25 | -4.90278 | -9.52259 | -1.43381 | 0.016 |
|  | 400 m walking time | p50 | -3.67473 | -5.27695 | -1.18576 | 0.096 |
|  | 400 m walking time | p75 | -4.08601 | -7.09224 | -0.70412 | 0.069 |
|  | Relative leg extension 1RM | p25 | -0.00019 | -0.01547 | 0.02268 | 0.987 |
|  | Relative leg extension 1RM | p50 | 0.01174 | -0.01161 | 0.02899 | 0.355 |
|  | Relative leg extension 1RM | p75 | 0.01405 | -0.00664 | 0.02272 | 0.276 |
|  | Handgrip strength | p25 | 0.62224 | -0.64625 | 1.80196 | 0.312 |
|  | Handgrip strength | p50 | 0.74459 | -0.17162 | 1.38979 | 0.236 |
|  | Handgrip strength | p75 | 0.89056 | -0.42254 | 2.15913 | 0.198 |
|  | Relative leg press 1RM | p25 | 0.03126 | -0.25818 | 0.05066 | 0.388 |
|  | Relative leg press 1RM | p50 | 0.0194 | -0.00324 | 0.05154 | 0.641 |
|  | Relative leg press 1RM | p75 | 0.01358 | -0.03826 | 0.19401 | 0.747 |
|  | Relative chest press 1RM | p25 | 0.01333 | -0.04538 | 0.02566 | 0.435 |
|  | Relative chest press 1RM | p50 | -0.00302 | -0.01426 | 0.05262 | 0.884 |
|  | Relative chest press 1RM | p75 | 0.03766 | -0.01817 | 0.17795 | 0.271 |
|  | Relative seated row 1RM | p25 | 0.00541 | -0.09897 | 0.04187 | 0.786 |
|  | Relative seated row 1RM | p50 | -0.00126 | -0.02009 | 0.0294 | 0.952 |
|  | Relative seated row 1RM | p75 | 0.00645 | 0.00279 | 0.05824 | 0.718 |
| 8 | Relative VO_2_peak | p25 | 0.48356 | 0.17877 | 0.819 | 0.042 |
|  | Relative VO_2_peak | p50 | 0.3799 | 0.17136 | 0.94323 | 0.105 |
|  | Relative VO_2_peak | p75 | 0.67542 | 0.345 | 1.11423 | 0.001 |
|  | Relative W_max_ | p25 | 0.04988 | 0.01827 | 0.08313 | 0.005 |
|  | Relative W_max_ | p50 | 0.05268 | 0.03426 | 0.09532 | 0.009 |
|  | Relative W_max_ | p75 | 0.07014 | 0.02933 | 0.12056 | 0.004 |
|  | 400 m walking time | p25 | -2.55856 | -9.62069 | -1.4592 | 0.129 |
|  | 400 m walking time | p50 | -3.64899 | -5.08336 | -0.79185 | 0.055 |
|  | 400 m walking time | p75 | -4.04181 | -6.23666 | -1.08631 | 0.029 |
|  | Relative leg extension 1RM | p25 | -0.00019 | -0.01567 | 0.02432 | 0.988 |
|  | Relative leg extension 1RM | p50 | 0.01165 | -0.01161 | 0.03002 | 0.365 |
|  | Relative leg extension 1RM | p75 | 0.01405 | -0.00708 | 0.02293 | 0.278 |
|  | Handgrip strength | p25 | 0.62224 | -0.64625 | 1.80196 | 0.312 |
|  | Handgrip strength | p50 | 0.74459 | -0.17162 | 1.38979 | 0.236 |
|  | Handgrip strength | p75 | 0.89056 | -0.42254 | 2.15913 | 0.198 |
|  | Relative leg press 1RM | p25 | 0.00084 | -0.15855 | 0.03443 | 0.977 |
|  | Relative leg press 1RM | p50 | 0.01294 | -0.05251 | 0.06964 | 0.776 |
|  | Relative leg press 1RM | p75 | -0.01216 | -0.0798 | 0.11551 | 0.738 |
|  | Relative chest press 1RM | p25 | 0.01124 | -0.01616 | 0.01605 | 0.315 |
|  | Relative chest press 1RM | p50 | 0.00997 | -0.01324 | 0.03636 | 0.512 |
|  | Relative chest press 1RM | p75 | 0.03366 | -0.00852 | 0.14045 | 0.255 |
|  | Relative seated row 1RM | p25 | -0.0032 | -0.10342 | 0.00379 | 0.839 |
|  | Relative seated row 1RM | p50 | -0.01132 | -0.01838 | 0.0241 | 0.527 |
|  | Relative seated row 1RM | p75 | 0.00517 | -0.03055 | 0.05341 | 0.780 |
| 10 | Relative VO_2_peak | p25 | 0.53151 | 0.20458 | 0.84154 | 0.021 |
|  | Relative VO_2_peak | p50 | 0.48117 | 0.22024 | 0.94046 | 0.034 |
|  | Relative VO_2_peak | p75 | 0.65555 | 0.32328 | 1.10346 | 0.001 |
|  | Relative W_max_ | p25 | 0.05101 | 0.02053 | 0.08224 | 0.003 |
|  | Relative W_max_ | p50 | 0.05268 | 0.0329 | 0.09557 | 0.009 |
|  | Relative W_max_ | p75 | 0.07014 | 0.02932 | 0.12011 | 0.004 |
|  | 400 m walking time | p25 | -2.41823 | -9.64623 | -1.3761 | 0.153 |
|  | 400 m walking time | p50 | -3.58205 | -5.3211 | -0.84799 | 0.068 |
|  | 400 m walking time | p75 | -4.38865 | -6.98541 | -1.06815 | 0.021 |
|  | Relative leg extension 1RM | p25 | -0.00019 | -0.01547 | 0.02268 | 0.987 |
|  | Relative leg extension 1RM | p50 | 0.01174 | -0.01161 | 0.02899 | 0.355 |
|  | Relative leg extension 1RM | p75 | 0.01405 | -0.00664 | 0.02272 | 0.276 |
|  | Handgrip strength | p25 | 0.62224 | -0.64625 | 1.80196 | 0.312 |
|  | Handgrip strength | p50 | 0.74459 | -0.17162 | 1.38979 | 0.236 |
|  | Handgrip strength | p75 | 0.89056 | -0.42254 | 2.15913 | 0.198 |
|  | Relative leg press 1RM | p25 | 0.00864 | -0.19384 | 0.04548 | 0.785 |
|  | Relative leg press 1RM | p50 | 0.01448 | -0.06082 | 0.07899 | 0.743 |
|  | Relative leg press 1RM | p75 | -0.01216 | -0.06488 | 0.07628 | 0.742 |
|  | Relative chest press 1RM | p25 | 0.01203 | -0.04531 | 0.0193 | 0.258 |
|  | Relative chest press 1RM | p50 | 0.00862 | -0.01047 | 0.03229 | 0.532 |
|  | Relative chest press 1RM | p75 | 0.03526 | -0.00505 | 0.14494 | 0.271 |
|  | Relative seated row 1RM | p25 | -0.00307 | -0.09571 | 0.01874 | 0.849 |
|  | Relative seated row 1RM | p50 | -0.00629 | -0.01849 | 0.02457 | 0.737 |
|  | Relative seated row 1RM | p75 | 0.00593 | -0.02895 | 0.05835 | 0.747 |
| 11 | Relative VO_2_peak | p25 | 0.53513 | 0.20544 | 0.83145 | 0.021 |
|  | Relative VO_2_peak | p50 | 0.45964 | 0.19099 | 0.96421 | 0.041 |
|  | Relative VO_2_peak | p75 | 0.67337 | 0.32618 | 1.0434 | 0.001 |
|  | Relative W_max_ | p25 | 0.05167 | 0.02072 | 0.0835 | 0.003 |
|  | Relative W_max_ | p50 | 0.05161 | 0.03538 | 0.09351 | 0.010 |
|  | Relative W_max_ | p75 | 0.07033 | 0.02943 | 0.1207 | 0.004 |
|  | 400 m walking time | p25 | -3.96602 | -9.50188 | -1.36742 | 0.031 |
|  | 400 m walking time | p50 | -3.68641 | -5.31056 | -1.03916 | 0.061 |
|  | 400 m walking time | p75 | -3.77475 | -6.8353 | -1.9229 | 0.037 |
|  | Relative leg extension 1RM | p25 | 0.00022 | -0.01409 | 0.02044 | 0.986 |
|  | Relative leg extension 1RM | p50 | 0.01188 | -0.01102 | 0.0296 | 0.351 |
|  | Relative leg extension 1RM | p75 | 0.01488 | -0.00654 | 0.02296 | 0.253 |
|  | Handgrip strength | p25 | 0.62224 | -0.64625 | 1.80196 | 0.312 |
|  | Handgrip strength | p50 | 0.74459 | -0.17162 | 1.38979 | 0.236 |
|  | Handgrip strength | p75 | 0.89056 | -0.42254 | 2.15913 | 0.198 |
|  | Relative leg press 1RM | p25 | 0.00864 | -0.18992 | 0.04469 | 0.788 |
|  | Relative leg press 1RM | p50 | 0.01488 | -0.05194 | 0.09596 | 0.752 |
|  | Relative leg press 1RM | p75 | -0.01525 | -0.07443 | 0.12209 | 0.694 |
|  | Relative chest press 1RM | p25 | 0.01475 | -0.0155 | 0.02272 | 0.188 |
|  | Relative chest press 1RM | p50 | 0.01079 | -0.00947 | 0.04436 | 0.395 |
|  | Relative chest press 1RM | p75 | 0.03291 | -0.00129 | 0.15502 | 0.266 |
|  | Relative seated row 1RM | p25 | -0.00307 | -0.09377 | 0.01889 | 0.845 |
|  | Relative seated row 1RM | p50 | -0.0022 | -0.01851 | 0.03263 | 0.906 |
|  | Relative seated row 1RM | p75 | 0.00593 | -0.0246 | 0.05522 | 0.748 |
| 12 | Relative VO_2_peak | p25 | 0.53147 | 0.18882 | 0.84515 | 0.022 |
|  | Relative VO_2_peak | p50 | 0.38956 | 0.20243 | 0.96232 | 0.094 |
|  | Relative VO_2_peak | p75 | 0.66082 | 0.32819 | 1.06795 | 0.001 |
|  | Relative W_max_ | p25 | 0.0499 | 0.01753 | 0.08285 | 0.004 |
|  | Relative W_max_ | p50 | 0.05111 | 0.03225 | 0.08666 | 0.010 |
|  | Relative W_max_ | p75 | 0.07014 | 0.02937 | 0.1198 | 0.004 |
|  | 400 m walking time | p25 | -2.45853 | -9.5425 | -1.37095 | 0.142 |
|  | 400 m walking time | p50 | -3.64111 | -5.135 | -0.9253 | 0.065 |
|  | 400 m walking time | p75 | -3.77475 | -6.2023 | -0.6868 | 0.037 |
|  | Relative leg extension 1RM | p25 | -0.00109 | -0.01836 | 0.01984 | 0.929 |
|  | Relative leg extension 1RM | p50 | 0.01214 | -0.01262 | 0.02755 | 0.335 |
|  | Relative leg extension 1RM | p75 | 0.01448 | -0.00623 | 0.02303 | 0.259 |
|  | Handgrip strength | p25 | 0.62224 | -0.64625 | 1.80196 | 0.312 |
|  | Handgrip strength | p50 | 0.74459 | -0.17162 | 1.38979 | 0.236 |
|  | Handgrip strength | p75 | 0.89056 | -0.42254 | 2.15913 | 0.198 |
|  | Relative leg press 1RM | p25 | 0.00691 | -0.23035 | 0.04871 | 0.832 |
|  | Relative leg press 1RM | p50 | 0.01297 | -0.06011 | 0.0616 | 0.772 |
|  | Relative leg press 1RM | p75 | -0.01354 | -0.07207 | 0.08203 | 0.733 |
|  | Relative chest press 1RM | p25 | 0.01203 | -0.0381 | 0.01936 | 0.275 |
|  | Relative chest press 1RM | p50 | 0.00546 | -0.01282 | 0.03562 | 0.682 |
|  | Relative chest press 1RM | p75 | 0.03526 | -0.00853 | 0.13814 | 0.272 |
|  | Relative seated row 1RM | p25 | -0.00418 | -0.11579 | 0.01568 | 0.780 |
|  | Relative seated row 1RM | p50 | -0.00629 | -0.01854 | 0.01492 | 0.739 |
|  | Relative seated row 1RM | p75 | 0.00645 | -0.02959 | 0.05423 | 0.732 |
| 13 | Relative VO_2_peak | p25 | 0.53824 | 0.20112 | 0.84226 | 0.020 |
|  | Relative VO_2_peak | p50 | 0.64969 | 0.17681 | 0.96229 | 0.003 |
|  | Relative VO_2_peak | p75 | 0.65747 | 0.38832 | 1.22056 | 0.001 |
|  | Relative W_max_ | p25 | 0.05404 | 0.02083 | 0.0836 | 0.002 |
|  | Relative W_max_ | p50 | 0.05326 | 0.04183 | 0.09353 | 0.008 |
|  | Relative W_max_ | p75 | 0.07084 | 0.02869 | 0.12596 | 0.004 |
|  | 400 m walking time | p25 | -3.57324 | -9.89638 | -1.57842 | 0.046 |
|  | 400 m walking time | p50 | -3.80517 | -5.28021 | -0.74985 | 0.051 |
|  | 400 m walking time | p75 | -4.26382 | -7.23097 | -2.2369 | 0.020 |
|  | Relative leg extension 1RM | p25 | 0.00046 | -0.01743 | 0.02341 | 0.970 |
|  | Relative leg extension 1RM | p50 | 0.01357 | -0.01248 | 0.02344 | 0.287 |
|  | Relative leg extension 1RM | p75 | 0.00717 | -0.00105 | 0.02258 | 0.556 |
|  | Handgrip strength | p25 | 0.62224 | -0.64625 | 1.80196 | 0.312 |
|  | Handgrip strength | p50 | 0.74459 | -0.17162 | 1.38979 | 0.236 |
|  | Handgrip strength | p75 | 0.89056 | -0.42254 | 2.15913 | 0.198 |
|  | Relative leg press 1RM | p25 | 0.00907 | -0.20737 | 0.04092 | 0.754 |
|  | Relative leg press 1RM | p50 | 0.00644 | -0.04064 | 0.08966 | 0.865 |
|  | Relative leg press 1RM | p75 | 0.00423 | -0.05332 | 0.14978 | 0.910 |
|  | Relative chest press 1RM | p25 | 0.01208 | -0.06379 | 0.01744 | 0.304 |
|  | Relative chest press 1RM | p50 | 0.00787 | -0.00694 | 0.05076 | 0.496 |
|  | Relative chest press 1RM | p75 | 0.03526 | -0.00879 | 0.15222 | 0.284 |
|  | Relative seated row 1RM | p25 | -0.00381 | -0.11855 | 0.01613 | 0.821 |
|  | Relative seated row 1RM | p50 | -0.01123 | -0.02143 | 0.04239 | 0.564 |
|  | Relative seated row 1RM | p75 | 0.00693 | -0.02878 | 0.07023 | 0.729 |
| 14 | Relative VO_2_peak | p25 | 0.53164 | 0.18282 | 0.83971 | 0.033 |
|  | Relative VO_2_peak | p50 | 0.42423 | 0.18385 | 1.15191 | 0.081 |
|  | Relative VO_2_peak | p75 | 0.69131 | 0.30735 | 1.1198 | 0.004 |
|  | Relative W_max_ | p25 | 0.05452 | 0.03167 | 0.08844 | 0.008 |
|  | Relative W_max_ | p50 | 0.05207 | 0.03239 | 0.09687 | 0.022 |
|  | Relative W_max_ | p75 | 0.07087 | 0.03335 | 0.12815 | 0.004 |
|  | 400 m walking time | p25 | -2.34523 | -6.50468 | -1.12032 | 0.157 |
|  | 400 m walking time | p50 | -3.24473 | -5.18154 | 0.26527 | 0.081 |
|  | 400 m walking time | p75 | -4.85361 | -6.83101 | -1.86961 | 0.008 |
|  | Relative leg extension 1RM | p25 | 0.01597 | 0.0033 | 0.03258 | 0.118 |
|  | Relative leg extension 1RM | p50 | 0.01822 | 0.00247 | 0.03676 | 0.116 |
|  | Relative leg extension 1RM | p75 | 0.0191 | -0.00446 | 0.02394 | 0.105 |
|  | Handgrip strength | p25 | 0.62224 | -0.64625 | 1.80196 | 0.312 |
|  | Handgrip strength | p50 | 0.74459 | -0.17162 | 1.38979 | 0.236 |
|  | Handgrip strength | p75 | 0.89056 | -0.42254 | 2.15913 | 0.198 |
|  | Relative leg press 1RM | p25 | 0.00864 | -0.19981 | 0.05328 | 0.785 |
|  | Relative leg press 1RM | p50 | 0.01623 | -0.05896 | 0.10008 | 0.715 |
|  | Relative leg press 1RM | p75 | -0.01354 | -0.05255 | 0.14165 | 0.735 |
|  | Relative chest press 1RM | p25 | 0.0119 | -0.03525 | 0.01965 | 0.231 |
|  | Relative chest press 1RM | p50 | 0.0054 | -0.00432 | 0.03536 | 0.656 |
|  | Relative chest press 1RM | p75 | 0.02284 | -0.00072 | 0.1293 | 0.251 |
|  | Relative seated row 1RM | p25 | -0.00307 | -0.07612 | 0.02298 | 0.857 |
|  | Relative seated row 1RM | p50 | -0.0024 | -0.01813 | 0.03278 | 0.901 |
|  | Relative seated row 1RM | p75 | 0.00645 | -0.03127 | 0.05792 | 0.739 |

All models were adjusted for age, body mass index, prostate cancer stage, time since diagnosis, and time on androgen deprivation therapy.

Abbreviations: 1RM, one-repetition maximum; β, unstandardised regression coefficient; CI, confidence interval; MVPA, moderate-to-vigorous physical activity; VO_2_peak, peak oxygen consumption; W_max_, maximal workload.

MVPA was analysed in hours∙week^-1^ to provide more interpretable β coefficients.

Participants with relative leg extension 1RM data: all participants, n = 115; ARI users, n = 44; non-users, n = 71.

Participants with relative leg press 1RM data: all participants, n = 49; ARI users, n = 18; non-users, n = 31.

Participants with relative chest press 1RM data: all participants, n = 51; ARI users, n = 21; non-users, n = 30.

Participants with relative seated row 1RM data: all participants, n = 48; ARI users, n = 21; non-users, n = 27.

Participants with handgrip strength data: all participants, n = 57; ARI users, n = 24, non-users, n = 33.
